# Formation and function of OmpG or OmpA-incorporated liposomes using an in vitro translation system

**DOI:** 10.1038/s41598-022-06314-4

**Published:** 2022-02-11

**Authors:** Koki Kamiya

**Affiliations:** Division of Molecular Science, Graduate School of Science and Technology, Gunma University, 1-5-1 Tenjin-cho, Kiryu, Gunma 376-8515 Japan

**Keywords:** Biophysical chemistry, Ion channels, Lipids, Membrane biophysics

## Abstract

Outer membrane proteins (OMPs), located on the outer membrane of gram-negative bacteria, have a β-strand structure and form nanopores, which allow passage of ions, sugars, and small molecules. Recently, OMPs have been used as sensing elements to detect biological molecules. OMPs are normally expressed and purified from *Escherichia coli *(*E. coli*). Although the cell-free synthesis of OMPs, such as OmpA and OmpG, is achieved in the presence of liposomes and periplasmic chaperones, the amount of OmpA and OmpG incorporated into the nano-sized liposomes is not clear. In this study, after in vitro translation, the incorporation of OmpG into purified nano-sized liposomes with various lipid compositions was investigated. Liposomes containing the synthesized OmpG were purified using a stepwise sucrose density gradient. We report that liposomes prepared with the *E. coli* lipid extract (PE/PG) had the highest amount of OmpG incorporated compared to liposomes with other lipid compositions, as detected by recording the current across the OmpG containing liposomes using the patch clamp technique. This study reveals some of the requirements for the insertion and refolding of OMPs synthesized by the in vitro translation system into lipid membranes, which plays a role in the biological sensing of various molecules.

## Introduction

Outer membrane proteins (OMPs) are inserted into the outer membrane of gram-negative bacteria^1,2^. OMPs with 8–26 β strands can assemble into β-barrels that can form nanopores in the membrane, where the number of β-strands determine the diameter of the nanopores^3,4^. Nanopores with varied diameters can allow passage of ions, sugars, and small molecules. Recently, OMPs are embedded into artificial cell membranes as sensing elements for detecting biological molecules using the patch-clamp technique, where biomolecules are detected based on the changes in the amplitude of current as they pass through the OMPs^5,6^. Therefore, OMPs are important not only for biological functions but also for biotechnological applications. OMPs are normally expressed and purified from *Escherichia coli *(*E. coli*)^7–10^ or are synthesized in an in vitro translation system^11,12^. The in vitro translation system has some advantages, including rapid expression and purification, when compared to the *E. coli.* expression system. Proteins with various characteristics, including those with different sizes or water-solubility, such as green fluorescent protein (GFP), connexin43, and human voltage-dependent anionic channel (hVDAC1), are synthesized using an in vitro translation system^13–16^. Functional membrane proteins can be purified using the in vitro translation solution, that includes the nano-sized liposomes, as the membrane proteins are inserted directly into the liposomes during membrane protein synthesis^14^. The lipid membrane insertion of OmpA and OmpG using the in vitro translation system is investigated based on the composition of the liposomes and the presence of periplasmic chaperones such as Skp, DegP, and SurA^11,12^. Although the synthesis of OmpA and OmpG using the in vitro translation system is carried out in the co-presence of the liposomes and the periplasmic chaperones, the exact amount of OmpA and OmpG that is incorporated in the nano-sized liposomes has not been clear because the liposome solution still contained the unincorporated proteins and the periplasmic chaperones, which were not purified after the in vitro translation^11,12^. The dynamics of the nanopore formation from OMPs synthesized by in vitro translation is also not clear.

**Figure 1:**
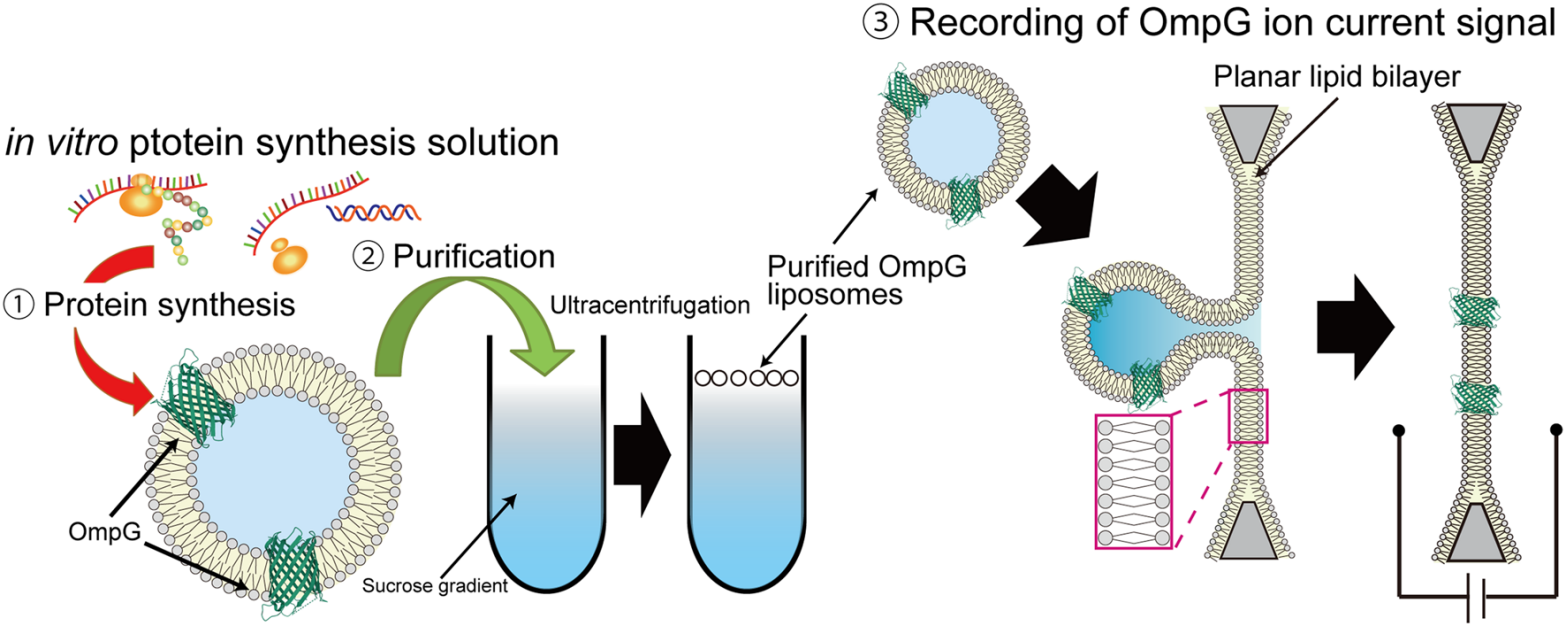
Schematic representation of formation and function of OmpG liposomes using the in vitro translation system.

In this study, the incorporation of OmpG into nano-sized liposomes, containing various lipid compositions, is investigated using purified nano-sized liposomes after in vitro translation (Fig. 1). OmpG contains an antiparallel β-barrel structure consisting of 14 transmembrane strands^17^. OmpG reconstituted into a bilayer lipid membrane (BLM) are used as biomolecule sensing^6,18^. To evaluate the amount of OmpG incorporated into the nano-sized liposomes produced using the in vitro translation solution, the liposomes are purified by a stepwise sucrose density gradient. The efficiency of incorporation of OmpG is confirmed by changing the lipid components and studying the diameter of the ensuing nano-sized liposomes. The experiment aimed at investigating the incorporation of OmpA also explored the applicability of the results obtained from the incorporation of OmpG. Finally, to investigate the nanopore formation of OmpG synthesized by in vitro translation, the ion currents of OmpG, which is incorporated into a BLM by fusing between the OmpG liposome and the BLM, are measured in the artificial cell membranes using the patch clamp system.

## Results and discussion

### Expression of OmpG using in vitro translation system

**Figure 2:**
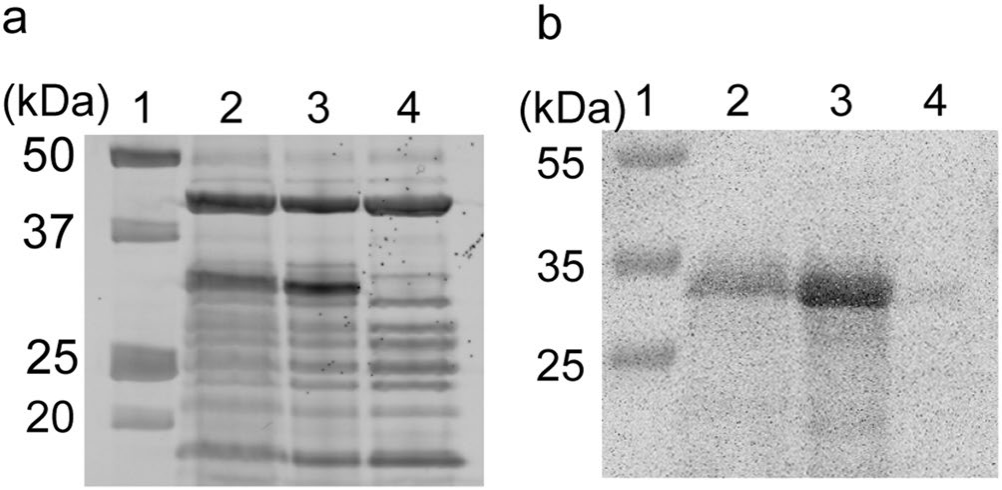
(**a**) SDS-PAGE analysis and (**b**) western blot analysis of OmpG synthesized by the in vitro translation system. Lane 1: protein molecular weight ladder, lane 2: Purefrex solution (+ OmpG encoded DNA) with nano-sized liposomes, lane 3: Purefrex solution (+ OmpG encoded DNA), lane 4: Purefrex solution (− OmpG encoded DNA).

OmpG, with or without addition of 20 mg/mL of a nano-sized liposome solution, was synthesized using a cell-free synthesis system. Figure 1 shows the results of the SDS-PAGE analysis and western blot analysis of the OmpG synthesis solution. A band of OmpG was observed at approximately 30 kDa, which was not observed in the solution of the in vitro translation system without OmpG DNA (Fig. 2a, Figure S1). The band of OmpG was detected by western blot analysis using an anti-His tag antibody because the His × 6 tag was conjugated at the N-terminal of OmpG (Fig. 2b, Figure S2). Although the amount of OmpG synthesized in solution with the nano-sized liposomes was lower than that without the nano-sized liposomes, a sufficient amount of OmpG in the solution with nano-sized liposomes was also synthesized.

### Incorporation of OmpG synthesized by the in vitro translation system into nano-sized liposomes

**Figure 3:**
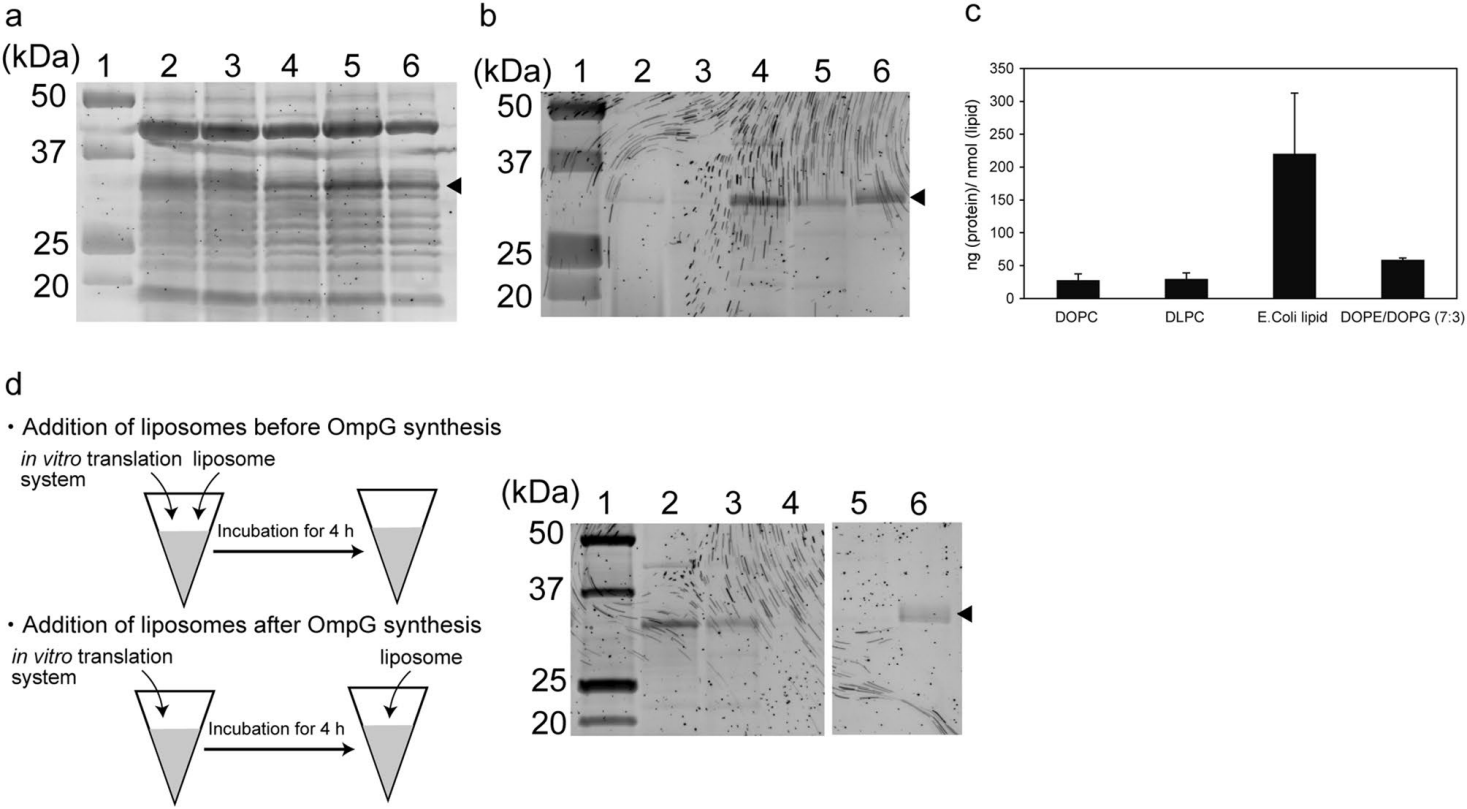
SDS-PAGE analysis of before (**a**) and after (**b**) stepwise sucrose density gradient purification of OmpG synthesis containing nano-sized liposomes. Lane 1: protein molecular weight ladder, lane 2: Purefrex solution (+ OmpG encoded DNA) with DOPC liposomes, lane 3: Purefrex solution (+ OmpG encoded DNA) with DLPC liposomes, lane 4: Purefrex solution (+ OmpG encoded DNA) with *Escherichia coli *(*E. coli*) lipid liposomes, lane 5: Purefrex solution (+ OmpG encoded DNA) with DOPE/DOPG liposomes, and (**a**) lane 6: Purefrex solution (+ OmpG encoded DNA) or (**b**) lane 6: purified OmpG expressed by *E. coli*. (**c**) Protein amount into nano-sized liposomes. (**d**) SDS-PAGE analysis of OmpG into nano-sized liposomes by addition of nano-sized liposomes before or after OmpG synthesis. Lane 1: protein molecular weight ladder, lane 2: Purefrex solution (+ OmpG encoded DNA) by adding *E. coli* lipid liposomes before OmpG synthesis, lane 3: Purefrex solution (+ OmpG encoded DNA) by adding DOPE/DOPG liposomes before OmpG synthesis, lane 4: Purefrex solution (+ OmpG encoded DNA) by adding *E. coli* lipid liposomes after OmpG synthesis, lane 5: Purefrex solution (+ OmpG encoded DNA) by adding DOPE/DOPG liposomes after OmpG synthesis, and lane 6: purified OmpG expressed by *E. coli*. Black triangles represent OmpG band. Although there is separation between lane1–4 and lane5,6, the same gel of lane1–6 was conducted.

To investigate whether OmpG synthesized using the in vitro translation system was directly incorporated into the nano-sized liposome membrane, OmpG was synthesized into a cell-free synthesis solution containing various liposome compositions (DOPC, DLPC, DOPE/DOPG (7:3wt/wt%), or *E. coli* polar lipid extract (PE/PG/CA (67:23.2:9.8 wt/wt%))). In previous study, reconstitution studies of the outer membrane proteins normally used phosphatidylcholines with various lengths of fatty acid chain (DHPC, DLPC, DMPC, DPPC, POPC, and DOPC) and phospholipid mixtures (DOPC/DOPE, and DMPC/DMPG)^11,12^. The membranes in *E. coli* are predominantly composed of phosphatidylglycerol (PG) and phosphatidylethanolamine (PE). Therefore, to compare the influences of the fatty acid chain and the phospholipid head groups, we selected these specific phospholipid compositions (DOPC, DLPC, DOPE/DOPG, and E. coli polar lipid extract). The nano-sized liposomes prepared by an extruder method were characterized by a dynamic light scattering method (Table 1). After incubation for 4 h at 37 °C, the incorporation of OmpG into the various liposome compositions was confirmed by SDS-PAGE. The bands of OmpG were detected in all samples containing nano-sized liposomes (Fig. 3a and Figure S3(a)). The synthesized amount of OmpG in the presence of the nano-sized liposomes is different from that of the lipid components of the nano-sized liposomes. However, the bands obtained from the samples comprised a mixture of bands representing the OmpG-incorporated liposomes, OmpG into the solution, and components of cell-free synthesis. Therefore, to confirm the incorporation of OmpG into liposomes, liposomes containing OmpG were separated from the synthesized samples using a sucrose density gradient centrifugation method. The lipid concentrations in the purified liposome samples were determined by measuring the concentrations of PC and PE. The bands of OmpG from the purified liposome compositions were detected by SDS-PAGE analysis when the liposome solution at a constant lipid concentration was applied to the SDS-PAGE gel. The OmpG band was only detected at approximately 30 kDa (Fig. 3b, Figure S3(b), Figure S4). These band positions of the OmpG, including the nano-sized liposomes, corresponded to the position of the band of the OmpG with the native conformation, suggesting that the OmpG, which is incorporated into nano-sized liposomes, is the folded OmpG^19^ (Fig. 3b, Figure S3(b)). The amount of OmpG incorporated into the nano-sized liposomes was as follows: large amounts of OmpG were incorporated into *E. coli* lipid liposomes, followed in order by DOPE/DOPG (7:3) liposomes, DOPC liposomes, and DLPC liposomes. The tendency of incorporation of OmpA into the nano-sized liposomes was the same tendency of the incorporation of OmpG into the nano-sized liposomes (Figure S5). We estimated protein amounts per a 1 nmol lipid from BCA protein assay kit. The protein amount of DOPC liposomes, DLPC liposomes, *E.coli* liposomes, and DOPE/DOPG (7:3) liposomes were 28.1 ± 9.3 (mean ± s.d.) ng (protein)/ nmol (lipid) (n = 2), 29.7 ± 9.3 (mean ± s.d.) ng (protein)/ nmol (lipid) (n = 2), 220.1 ± 92.5 (mean ± s.d.) ng (protein)/ nmol (lipid) (n = 2), 58.8 ± 2.9 (mean ± s.d.) ng (protein)/ nmol (lipid) (n = 2), respectively (Fig. 3c). The protein amount into these liposomes corresponded to these band intensities of the SDS-PAGE gel analysis. The zeta potential values (mean ± s.d.; n = 3) of DOPC liposomes, DLPC liposomes, *E.coli* liposomes, and DOPE/DOPG (7:3) liposomes were − 5.3 ± 0.2 mV, − 1.2 ± 0.1 mV, − 39.7 ± 2.6 mV, and − 55.2 ± 0.4 mV, respectively. The amount of OmpG incorporated into the DOPE/DOPG (7:3) liposomes was larger than that of the DOPC liposomes (Fig. 3b, Figure S3(b)). Although the length of the fatty acid chain in the DOPC and DOPE/DOPG liposomes is equivalent, the surface of DOPE/DOPG was more negative compared to that of the DOPC liposomes. On comparing the *E. coli* lipid liposomes and DOPE/DOPG (7:3) liposomes, a larger amount of OmpG was observed to be incorporated into the *E. coli* lipid liposome than into the DOPE/DOPG liposomes, even though the surface charge of the DOPE/DOPG liposomes was more negative compared to that of the *E. coli* lipid liposomes. The length of the fatty acid chain is different between the *E. coli* lipid liposome (containing fatty acid chains of various lengths) and DOPE/DOPG (containing fatty acid chains of a common length). These results indicate that the incorporation of OmpG is not only influenced by the amount of negative charge but also the variability in the length of the fatty acid chain of the liposomes. In previous studies involving OmpA and OmpG synthesis by the in vitro translation system, the SDS-PAGE analysis of the OmpA and OmpG-containing liposome solution that was not purified after the in vitro translation was found to be ineffective for estimating the amount of OmpA and OmpG incorporated into the nano-sized liposomes^11,12^. In this study, with the use of purified liposomes, the amount of OmpG incorporated in nano-sized liposomes, with various lipid components, was estimated for the first time. Although distinguishing between the OMPs embedded in liposomes and those present freely in the solution is difficult using previous methods, this experiment shows that the highest amount of OmpG was inserted into the *E. coli* lipid liposomes, which were in the folded state in the absence of periplasmic chaperones such as Skp, DegP, and SurA.

**Table 1:** Hydrodynamic diameter and polydispersity index of nano-sized liposomes.

Lipid compositions of nano-sized liposomes	DOPC	DLPC	*Escherichia coli *(*E. coli*) extract polar	DOPE/DOPG (7:3 wt/wt%)
Hydrodynamic diameter (nm)	214.9	340.2	145.6	193.1
Polydispersity index	0.284	0.254	0.150	0.175

Next, when the liposomes were added to the solution of the in vitro translation system before or after the synthesis of OmpG, the differences in the amount of OmpG incorporated into the *E. coli* lipid or DOPE/DOPG liposomes were investigated after the stepwise sucrose density gradient of the liposomes (Fig. 3d, Figure S3(b)). In case of the addition of the liposomes before the OmpG synthesis, the band of OmpG on the *E. coli* lipid or DOPE/DOPG liposomes was detected by SDS-PAGE analysis. In contrast, in the case of the addition of liposomes after OmpG synthesis, the band of OmpG on these liposomes was not detected. This result suggests that OmpG was incorporated into liposomes during OmpG synthesis. When membrane proteins were synthesized by the in vitro translation system, membrane proteins were incorporated into liposomes during their synthesis; in other words, the membrane proteins were aggregated in the absence of the liposomes during the cell-free synthesis^14^. Therefore, cell-free synthesis of functional membrane proteins requires the coexistence of liposomes. These results corresponded with previous results on membrane protein synthesis using an in vitro translation system^20^.

### Electrophysiological measurement of OmpG on the nano-sized liposomes

**Figure 4:**
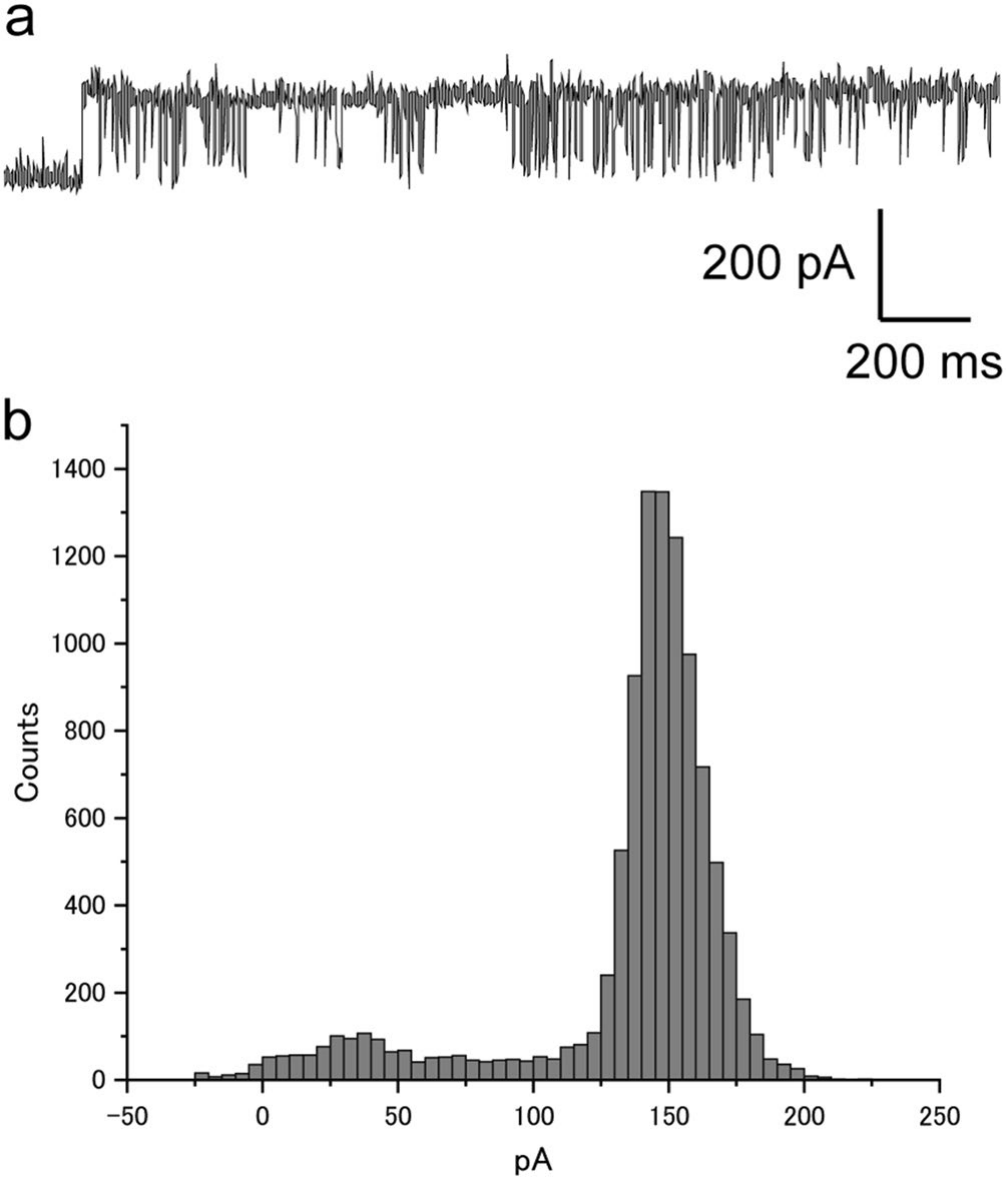
(**a**) Single channel current recording of OmpG incorporated into nano-sized liposomes (applied voltage: + 100 mV). (**b**) Histogram of OmpG current amplitude.

To confirm the formation of nanopores of OmpG into the nano-sized liposomes, the current signals of the OmpG were measured by a BLM chip that was connected to a patch clamp amplifier^21^. The BLM was formed using the droplet contact method^22^. OmpG-containing liposomes were initially added to the droplets. By the fusion of liposomes with BLM, OmpG was incorporated into the BLM. We obtained an OmpG current amplitude of approximately 150 pA at + 100 mV with 1 M KCl [Fig. 4a,b]. This amplitude was similar to that previously reported for OmpG channels in the BLM system^23^. This open-close signal shape is specific to OmpG. The in vitro translation system-synthesized OmpG without the nano-sized liposomes as a negative control obtained ion current signals measured by the patch clamp system. Outer membrane proteins lack a native conformation in the absence of liposomes or detergents.

Although the ion current signals from the OmpG samples without the nano-sized liposomes appeared (14 experiments), the ion current amplitude of OmpG without the nano-sized liposomes was not constant (Figure S6). From this ion current measurement of the single nanopore, some OmpGs having a native conformation were incorporated in into the nano-sized liposomes. The specific current amplitude of OmpA in the nanosized liposomes was also detected (Figure S6). To the best of our knowledge, this is the first report of the ion current detection of Omp synthesized using an in vitro translation system.

## Conclusion

In this study, we show that OmpG incorporated into nano-sized liposomes can be quantified by stepwise sucrose density gradient, which can remove the liposome-free OmpG and other molecules of the in vitro translation system from the OmpG-containing liposomes. Therefore, the amount of OmpG that is incorporated into the nano-sized liposomes when synthesized by the in vitro translation system changed with different lipid compositions, including the charge of the head group and the length of the acyl group. The liposomes produced with the *E. coli* lipid composition (PE/PG with various acyl group lengths) had the highest amount of OmpG incorporated compared to liposomes with other lipid compositions. The native conformation of OmpG incorporated into liposomes was revealed by patch-clamp analysis. This study reveals some of the requirements for the insertion and refolding of OMPs synthesized by the in vitro translation system into lipid membranes. The folded OmpG was easily obtained using the in vitro translation system. The OmpG-containing liposome plays a role in the biological sensing for detecting various molecules using OmpG nanopores.

## Materials and methods

### Reagents

1,2-Dioleoyl-*sn*-glycero-3-phosphocholine (DOPC), 1,2-dioleoyl-*sn*-glycero-3-phospho-(1’-rac-glycerol) (DOPG), 1,2-dioleoyl-*sn*-glycero-3-phosphoethanolamine (DOPE), 1,2-dilauroyl-*sn*-glycero-3-phosphocholine (DLPC), and *Escherichia coli *(*E. coli*) extract polar were purchased from Avanti Polar Lipids, Inc. (Alabaster, AL, USA). PURE*frex*2.0 was purchased from Cosmo Bio (Tokyo, Japan). A pre-cast gel of 15% SDS-polyacryamide gel was purchased from ATTO (Tokyo, Japan). HEPES was purchased from DOJINDO LABORATORIES (Kumamoto, Japan). KCl and sucrose were purchased from FUJIFILM Wako Chemicals (Tokyo, Japan). TaKaRa BCA protein assay kit was purchased from TaKaRa BIO (Shiga, Japan).

### Synthesis of OmpG using the in vitro translation system

OmpG was synthesized using the PURE*frex*2.0 system. Solution I (6.25 μL), solution II (0.625 µL), solution III (1.25 μL), OmpG or OmpA-coded DNA (1 µL; 0.5 mg/mL), and dH_2_O (3.375 µL) were added to the PCR tube. The mixture was incubated for 4 h at 37 °C.

### Synthesis and purification of OmpG containing the liposome solution using in vitro translation system

The lipids (DOPC, DLPC, DOPE/DOPG, and *E. coli* lipid) dissolved in chloroform were evaporated under flowing argon gas until lipid films formed at the bottom of the glass tube. The lipid films were hydrated by adding 50 mM HEPES (pH 7.4) and then vortexed. Next, 20 mg/mL liposomes were prepared. To obtain nano-sized liposomes, the liposome solution was filtered using an Avanti Mini-extruder with a polycarbonate membrane of 100 nm diameter apertures^24^. A total of eleven passes through the membrane was conducted at 20–25 °C. Hydrodynamic diameters and polydispersity index of 1 mg/mL nano-sized liposomes were measured using a dynamic scattering method (ELSZ-2000; Otsuka Electronics, Osaka, Japan). A data was obtained by repeating measurement (25 times). Zeta potential values of 5 mg/mL nano-sized liposomes were measured with a Zetasizer nano ZPS instrument (Malvern Instruments, United Kingdom). OmpG and OmpA were synthesized using PURE*frex*2.0 in the presence of nano-sized liposomes. A total of 12.5 μL of solution I, 1.25 μL of solution II, 2.5 μL of solution III, 2 µL of DNA, 6.75 μL of dH_2_O, and 10 µL of 20 mg/mL nano-sized liposomes were added to the PCR tube. The liposome concentration was determined as follow previous work^19^. The mixture was incubated for 4 h at 37 °C. The OmpG and OmpA-incorporated liposomes were separated using a stepwise sucrose density gradient [1.4 M, 0.8 M, and 0.5 M sucrose in 50 mM HEPES (pH 7.4)] at 40,000 rpm for 3 h at 4 °C (S52ST rotor, himac, Tokyo, Japan), and the OmpG- and OmpA-containing liposome fractions were collected. The protein concentration was measured using the TaKaRa BCA protein assay kit (TaKaRa BIO, Shiga, Japan). The phosphocholine (PC) concentration for DOPC and DLPC liposomes, and the phosphoethanolamine (PE) concentration for DOPE/DOPG and *E. coli* lipid liposomes were measured. Each liposome that was adjusted for the lipid concentrations was analyzed by electrophoresis. One volume of sample buffer (Laemmli Sample Buffer, BIO-RAD, Hercules, CA, USA) was added to the OmpG and OmpA liposome samples. These mixtures were separated on a 15% SDS-PAGE gel, which was immersed in Oriole Fluorescent Gel stain (BIO-RAD, Hercules, CA, USA)). The bands of OmpG and OmpA were detected using a gel imaging system (LuminoGraph I, ATTO, Tokyo, Japan).

### Electrophysiological measurement of OmpG

Eight microliters of 20 mg/mL DOPC solution dissolved in n-decane was added to each double well^10,21^. Next, 23 µL of buffer solution (10 mM HEPES/1 M KCl, pH 7.4) containing the OmpG liposomes was added to each well. The BLM spontaneously formed into an aperture diameter of 500 µm on a separator integrated into the double well. OmpG was incorporated into the BLM by fusing the liposomes and BLM. This double-well device was connected to a portable patch-clamp amplifier (pico2, Tecella, California, USA). The current signals of OmpG were recorded using a patch clamp amplifier with a 1 kHz low-pass filter at a sampling frequency of 5 kHz (Tecella JET, California, USA). The measurement temperature was 20–23 °C. The current signals were analyzed using the pCLAMP software program (Molecular Devices, California, USA).

## Supplementary Information

Supplementary Figures.
