# Supplementary material for: Formation and function of OmpG or OmpA-incorporated liposomes using an in vitro translation system
Source: Sci Rep. 2022 Feb 11;12:2376. doi: 10.1038/s41598-022-06314-4 (PMC8837779; doi:10.1038/s41598-022-06314-4)
Supplement: Supplementary file 1 — Supplementary Figures. [file 41598_2022_6314_MOESM1_ESM.docx]

**Electronic Supplementary Information**

**Formation and function of OmpG or OmpA-** **incorporated liposomes using an in vitro translation system**

**Koki Kamiya^1*^**

^1^ Division of Molecular Science, Graduate School of Science and Technology, Gunma University, 1-5-1 Tenjin-cho, Kiryu, Gunma 376-8515, Japan

*Corresponding author:

Koki Kamiya

Division of Molecular Science, Graduate School of Science and Technology, Gunma University, 1-5-1 Tenjin-cho, Kiryu, Gunma 376-8515, Japan

Phone: +81-277-30-1342

Fax: +81-277-30-1342

Email: kamiya@gunma-u.ac.jp


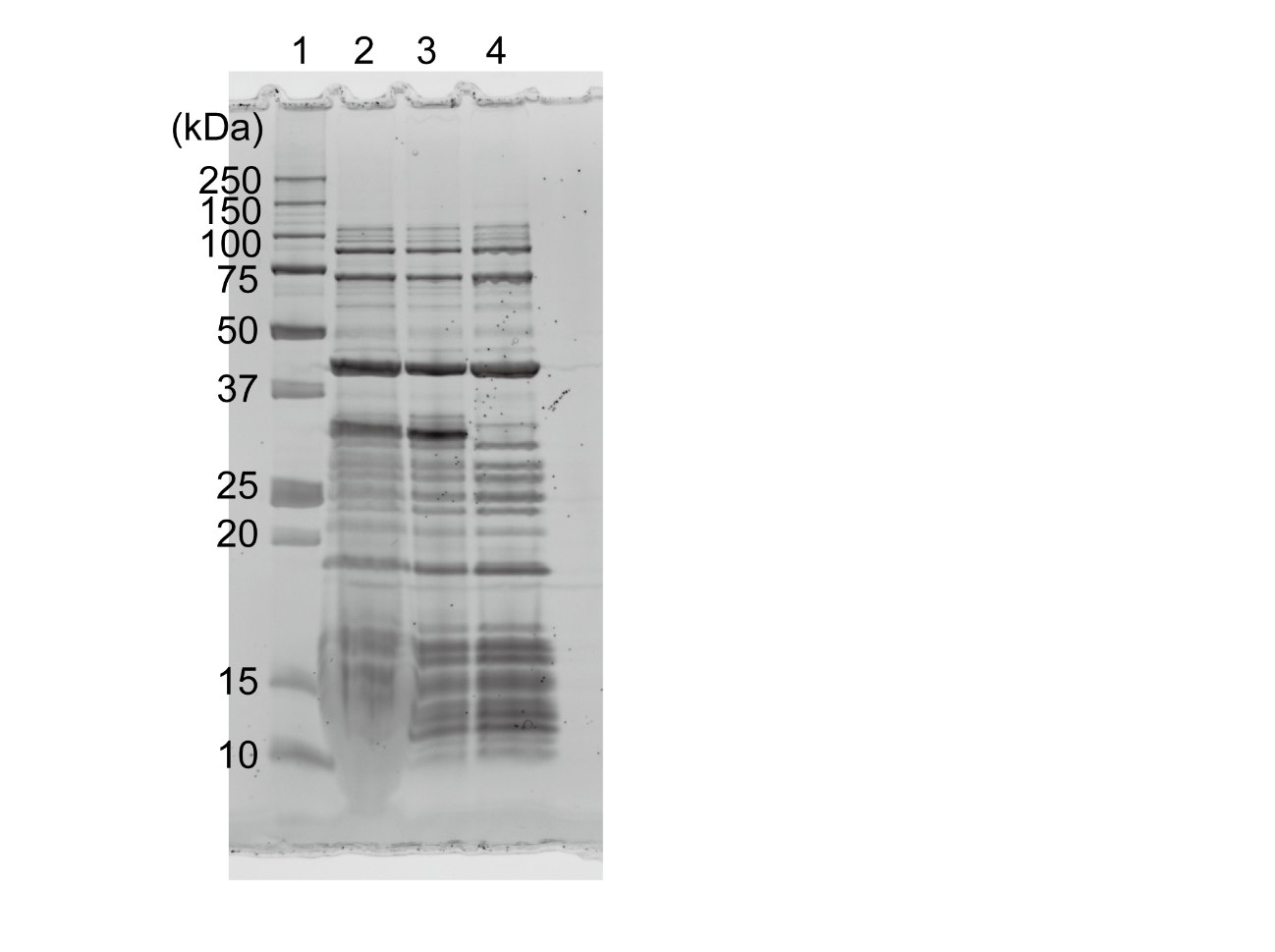


**Figure S1**. Full-length SDS-PAGE for Figure 2 (a).


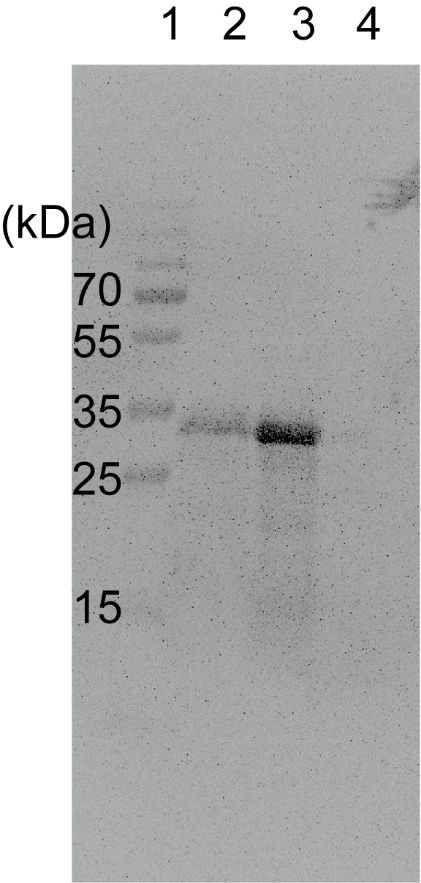


**Figure S2**. Full-length western blot for Figure 2 (b).


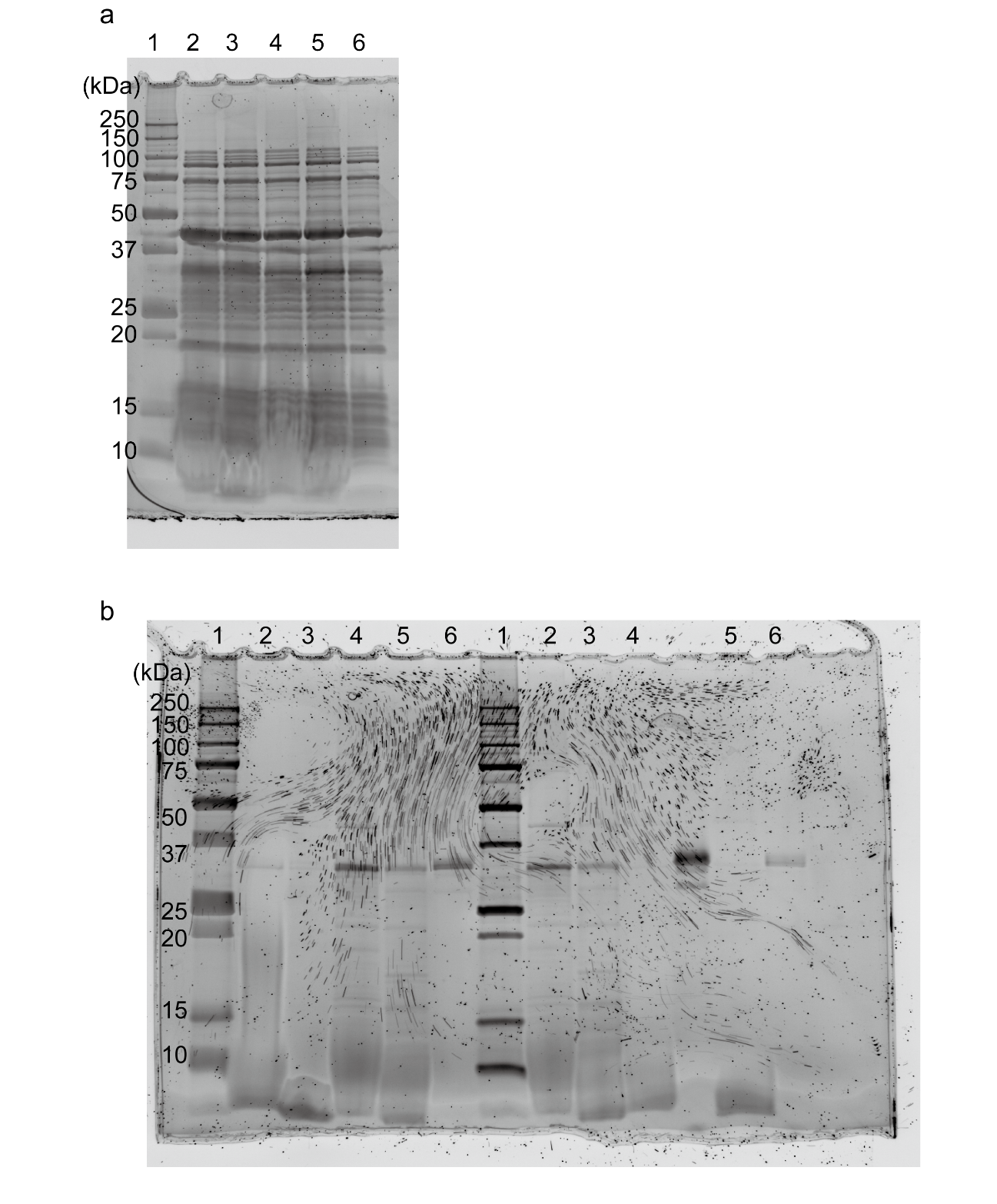


**Figure S3**. (a) Full-length SDS-PAGE for Figure 3 (a). (b) Full-length SDS-PAGE for Figure 3 (b) and (c).


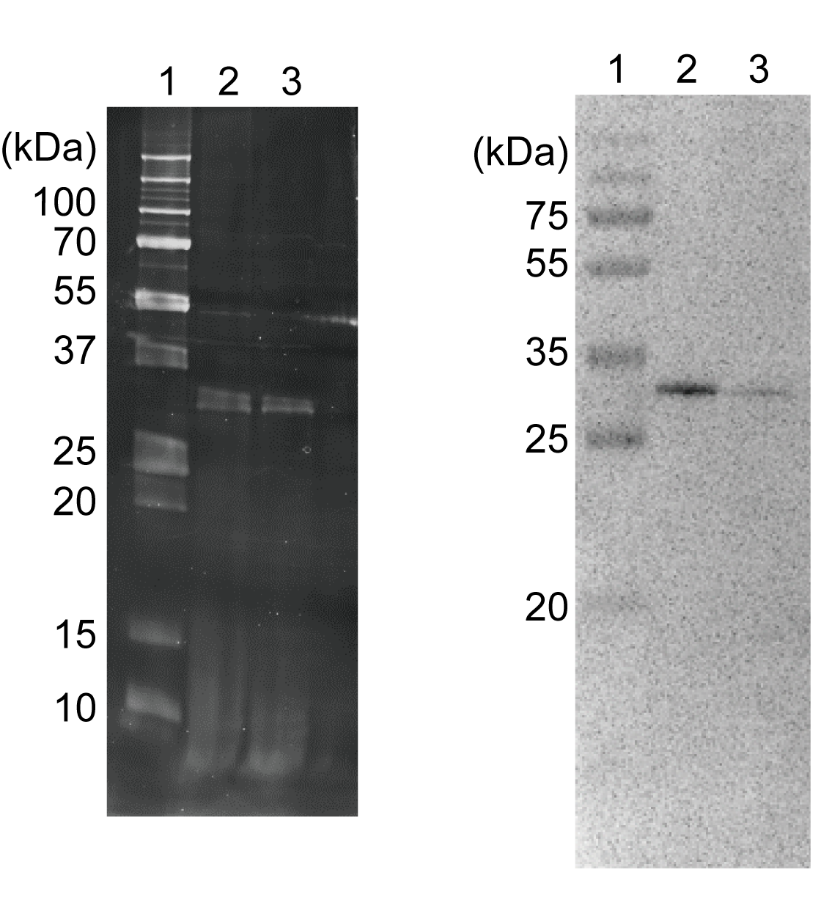


**Figure S4**. SDS-PAGE analysis (left) and western blot analysis (right) of after stepwise sucrose density gradient purification of OmpG synthesis containing nano-sized liposomes. Lane 1: protein molecular weight ladder, lane 2: Purefrex solution (+ OmpG encoded DNA) with E. coli lipid liposomes, and lane 3: Purefrex solution (+ OmpG encoded DNA) with DOPE/DOPG liposomes.


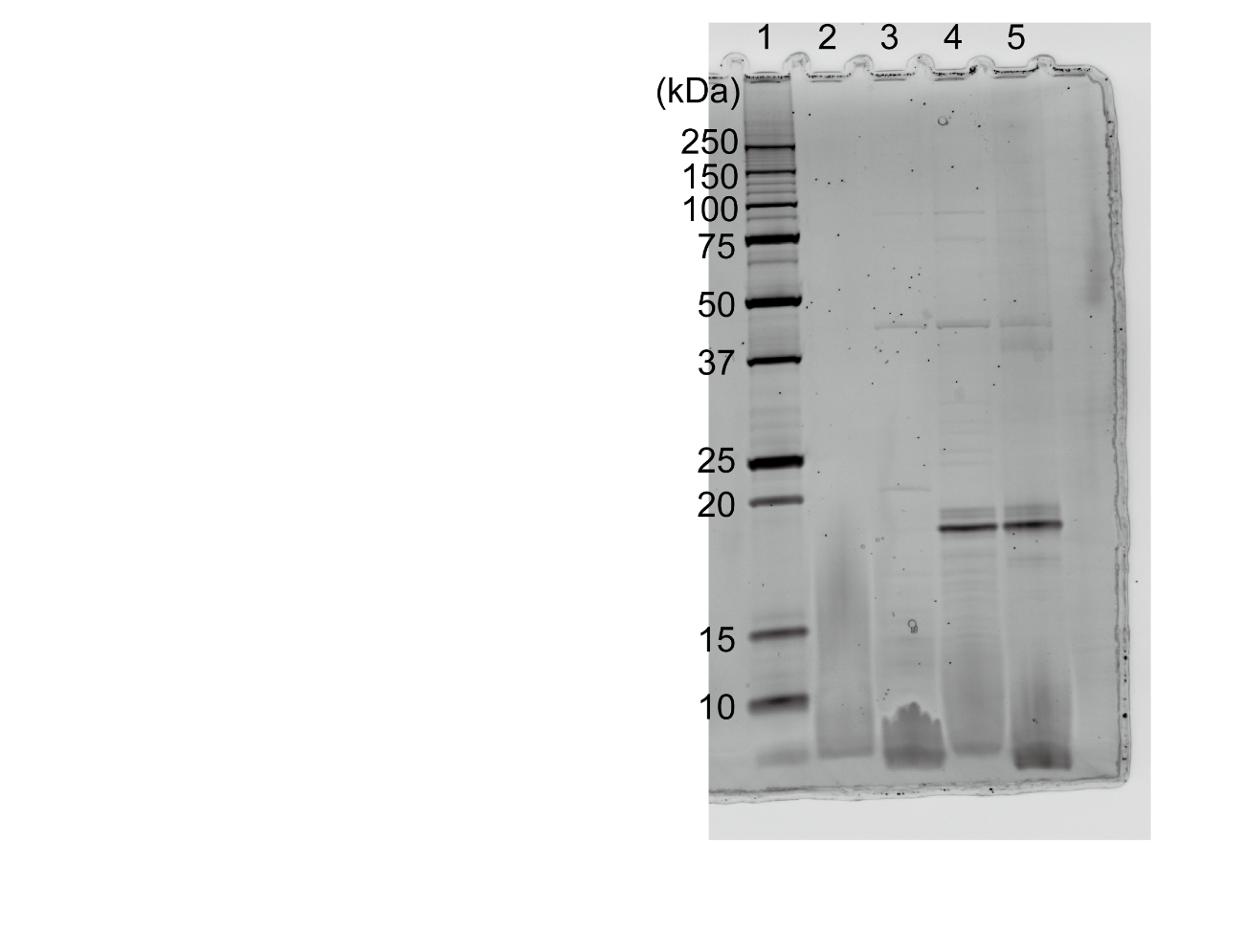


**Figure S5**. SDS-PAGE analysis of after stepwise sucrose density gradient purification of OmpA synthesis containing nano-sized liposomes. Lane 1: protein molecular weight ladder, lane 2: Purefrex solution (+ OmpA encoded DNA) with DOPC liposomes, lane 3: Purefrex solution (+ OmpA encoded DNA) with DLPC liposomes, lane 4: Purefrex solution (+ OmpA encoded DNA) with E. coli lipid liposomes, lane 5: Purefrex solution (+ OmpA encoded DNA) with DOPE/DOPG liposomes.


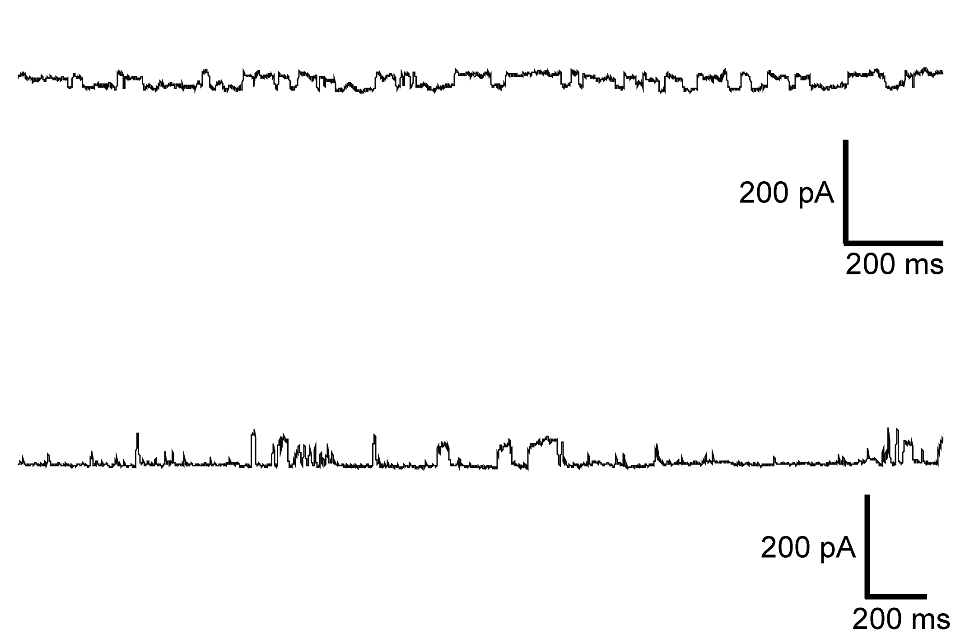


**Figure S6.** Ion current signal of OmpG sample without nano-sized liposomes as negative control (applied voltage: +100 mV).


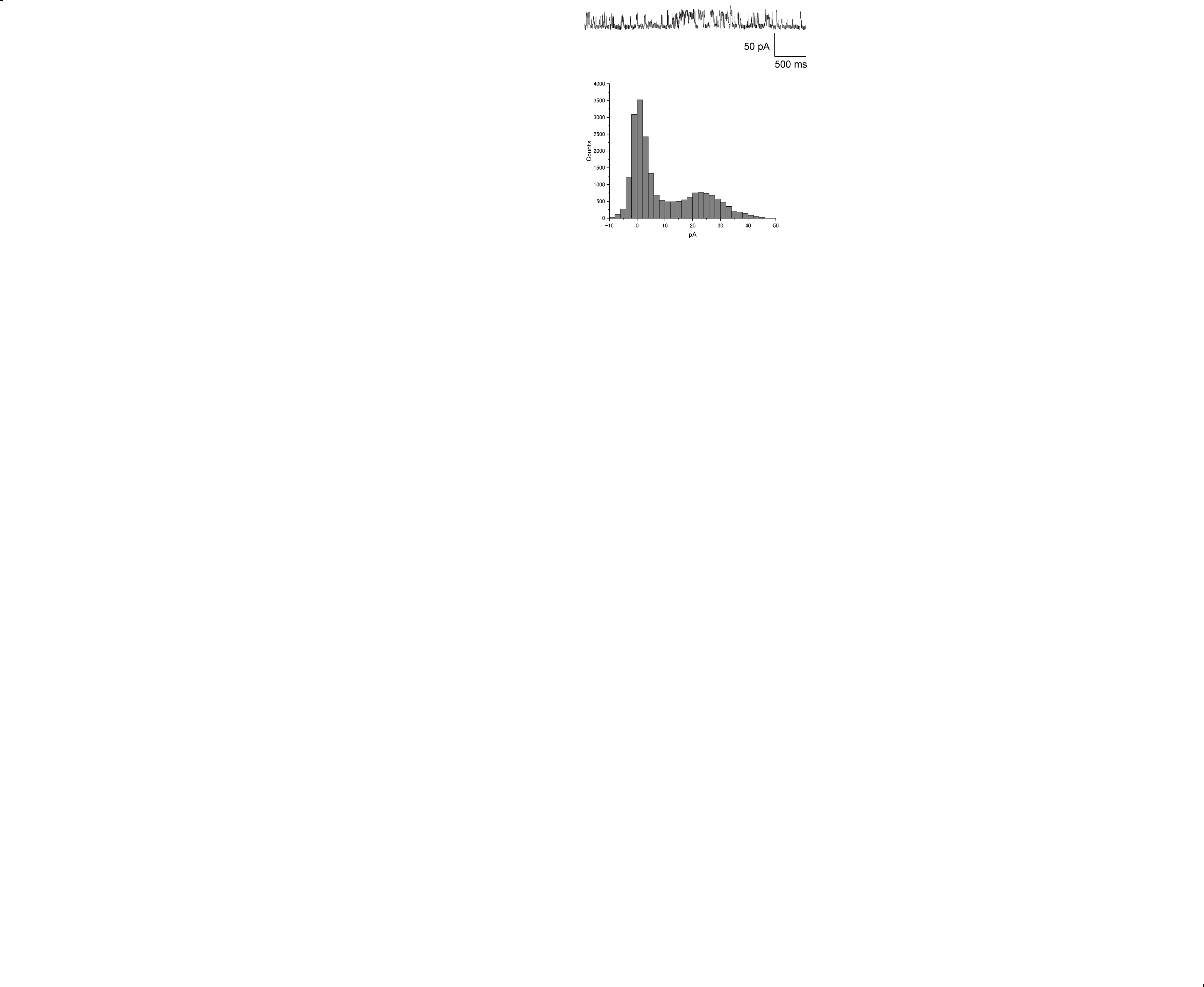


**Figure S7.** Single channel current recording of OmpA incorporated into nano-sized liposomes (applied voltage: +100 mV). Histogram of OmpA current amplitude.
